# Prevalence of insomnia and its association with quality of life among Macau residents shortly after the summer 2022 COVID-19 outbreak: A network analysis perspective

**DOI:** 10.3389/fpsyt.2023.1113122

**Published:** 2023-02-16

**Authors:** Pan Chen, Ling Zhang, Sha Sha, Mei Ieng Lam, Ka-In Lok, Ines Hang Iao Chow, Tong Leong Si, Zhaohui Su, Teris Cheung, Yuan Feng, Todd Jackson, Yu-Tao Xiang

**Affiliations:** ^1^Unit of Psychiatry, Department of Public Health and Medicinal Administration, and Institute of Translational Medicine, Faculty of Health Sciences, University of Macau, Macao, Macao SAR, China; ^2^Centre for Cognitive and Brain Sciences, University of Macau, Macao, Macao SAR, China; ^3^Beijing Key Laboratory of Mental Disorders, National Clinical Research Center for Mental Disorders, National Center for Mental Disorders, Beijing Anding Hospital, Capital Medical University & Advanced Innovation Center for Human Brain Protection, Capital Medical University, Beijing, China; ^4^Kiang Wu Nursing College of Macau, Macao, Macao SAR, China; ^5^School of Public Health, Southeast University, Nanjing, China; ^6^School of Nursing, The Hong Kong Polytechnic University, Hong Kong, Hong Kong SAR, China; ^7^Department of Psychology, University of Macau, Macao, Macao SAR, China

**Keywords:** insomnia, quality of life, COVID-19 outbreak, prevalence, network analysis

## Abstract

**Background:**

The latest wave of the coronavirus disease 2019 (COVID-19) pandemic in Macau began on 18 June 2022 and was more serious than previous waves. Ensuing disruption from the wave is likely to have had a variety of negative mental health consequences for Macau residents including increased risk for insomnia. This study investigated the prevalence and correlates of insomnia among Macau residents during this wave as well as its association with quality of life (QoL) from a network analysis perspective.

**Methods:**

A cross-sectional study was conducted between 26 July and 9 September 2022. Univariate and multivariate analyses explored correlates of insomnia. Analysis of covariance (ANCOVA) examined the relationship between insomnia and QoL. Network analysis assessed the structure of insomnia including “Expected influence” to identify central symptoms in the network, and the flow function to identify specific symptoms that were directly associated with QoL. Network stability was examined using a case-dropping bootstrap procedure.

**Results:**

A total of 1,008 Macau residents were included in this study. The overall prevalence of insomnia was 49.0% (*n* = 494; 95% CI = 45.9–52.1%). A binary logistic regression analysis indicated people with insomnia were more likely to report depression (OR = 1.237; *P* < 0.001) and anxiety symptoms (OR = 1.119; *P* < 0.001), as well as being quarantined during the COVID-19 pandemic (OR = 1.172; *P* = 0.034). An ANCOVA found people with insomnia had lower QoL (F_(1,1,008)_ = 17.45, *P* < 0.001). “Sleep maintenance” (ISI2), “Distress caused by the sleep difficulties” (ISI7) and “Interference with daytime functioning” (ISI5) were the most central symptoms in the insomnia network model, while “Sleep dissatisfaction” (ISI4), “Interference with daytime functioning” (ISI5), and “Distress caused by the sleep difficulties” (ISI7) had the strongest negative associations with QoL.

**Conclusion:**

The high prevalence of insomnia among Macau residents during the COVID-19 pandemic warrants attention. Being quarantined during the pandemic and having psychiatric problems were correlates of insomnia. Future research should target central symptoms and symptoms linked to QoL observed in our network models to improve insomnia and QoL.

## 1. Introduction

Since 2019, Coronavirus Disease 2019 (COVID-19) has been a constant threat that affects daily lives. Repeated and sporadic outbreaks of COVID-19 have brought unprecedented challenges worldwide. The Macau Special Administrative Region of China (Macao SAR) has had a unique geo-political position in the context of COVID-19 due to its heavy reliance upon mainland Chinese visitation and gambling revenues to support the local economy (1, 2). The latest wave of the COVID-19 pandemic in Macau occurred from 18 June to 7 August 2022. Labeled as the “618” pandemic, this wave resulted in the largest number of confirmed cases in the history of Macau, with 705 (38.7%) new confirmed cases and 1,116 (61.3%) asymptomatic infection cases (3). In response, the Macau SAR government took immediate, active, progressive measures that included temporary shut-down of entry/exit to mainland China, suspension of business and public institution operations (e.g., casinos, schools, construction sites), and self-isolation to maintain “relative static management” for 12 days from 11 July 2022 (3). The estimated number of visitors from mainland China decreased sharply from 336,488 in June, 2022 to 7,321 in July 2022 (4). A high perceived contagion risk during the wave increased negative emotions and curtailed willingness to travel (5). Additionally, ongoing border restrictions during the entire COVID-19 pandemic have restricted the flow of domestic and foreign tourists with devastating effects on the economy of Macau (6). Macau SAR has followed the “dynamic zero-COVID” prevention policy of mainland China that had been remarkably effective in minimizing the spread of COVID-19 (7, 8) but was accompanied by increases in potentially chronic psychological problems (9), such as depression, anxiety, and insomnia (10, 11).

One of the most common behavioral health consequences of COVID-19 and the dynamic zero policy has been insomnia, which is the most common sleep disorder and is characterized by difficulty initiating or maintaining sleep, accompanied by daytime impairments in functioning (12, 13). Insomnia is a risk factor for progression to or exacerbations of other physical and psychiatric disorders in addition to broader costs to social life and healthcare (13). Insomnia has also been a widespread health that poses significant challenges for prevention and treatment during the COVID-19 pandemic (14). A previous insomnia study revealed a pooled prevalence of 11.3% with significant differences across 13 countries before the COVID-19 pandemic (15). However, the rate of insomnia symptoms was up to 36.7% in a large multi-center survey conducted during the COVID-19 pandemic (16). During the initial COVID-19 outbreak, the insomnia rate among Macau residents was as high as 27.6% (17). However, given the increased severity of the most recent wave, the prevalence of insomnia in Macau should be re-estimated.

A variety of factors can contribute to insomnia amid pandemic conditions including concerns about infection and the rapidly increasing number of cases (18), social isolation or lockdown (19), and stress from economic hardships (14). Insomnia has been described as both a symptom and a sign of increased psychiatric morbidity, especially in relation to depression and anxiety disorders (15, 20). Sociodemographic correlates of insomnia have also been documented, though results are sometimes discrepant. For example, some studies suggested younger adults have more severe insomnia symptoms (21, 22) while other research has found insomnia is more common among older adults (15, 23). In addition, due to its potential chronicity, insomnia is associated with substantial impairments in quality of life (QoL) (15, 20, 24) and such losses are often an impetus for seeking treatment (25). However, to date, there has been an absence of studies on relevant correlates of insomnia among Macau residents or the nature of its links with QoL during this latest serious COVID-19 wave.

Network analysis (NA) provides a new perspective for exploring psychiatric problems that are conceptualized as causally linked symptom systems (26). A network is comprised of nodes that represent symptoms of a particular syndrome and edges that represent intercorrelations between symptoms (27). NA offers insight into how symptoms of a syndrome are interconnected and can be used to identify the most central or influential symptoms of a syndrome that can be prioritized as specific targets for interventions (28). NA has been applied widely to multiple psychiatric disorders among various subpopulations during the COVID-19 pandemic (29–31). A previous network model of insomnia symptoms among mental health professionals suggested that “interference with daytime functioning” was the most influential symptom (29). However, to date, no studies have focused on insomnia symptoms or their links with QoL among Macau residents during the recent 618 COVID-19 wave.

To address these gaps, this study (1) investigated the prevalence and correlates of insomnia among Macau residents during the “618” COVID-19 waves, (2) examined the most central symptoms within the network model of insomnia, and (3) evaluated relations between particular insomnia symptoms and QoL in the sample. We hypothesized that insomnia would be common and negatively associated with QoL among Macau residents in this wave.

## 2. Materials and methods

### 2.1. Participants and procedure

This cross-sectional study was conducted between 26 July and 9 September 2022 in Macau, China using a snowball sampling method. Due to lockdowns and the potential risk of infection during the wave, face-to-face interviews could not be performed. Following previous studies (17, 31–33), the study was conducted online using snowball convenience sampling and participants were invited to participate in this survey on reactions to COVID-19 using a Quick Response code (QR code) through advertisements in major social network platforms (specifically: WeChat, Facebook, and Instagram). The QR linked to an invitation and questionnaires. Inclusion criteria were as follows: (1) Residents who lived in Macau during the recent 618 COVID-19 wave; (2) aged 18 years or above; (3) able to understand the purposes and content of the survey. There were no specific exclusion criteria for this study. All participants provided electronic written informed consent on a voluntary and confidential basis. This study protocol was approved by the Institutional Review Board (IRB) of the University of Macau.

### 2.2. Measures

Basic socio-demographic information was collected including age, gender, marital status, living status, education level, and employment status during the COVID-19 pandemic. Additionally, several variables related to COVID-19 were measured, including concern about COVID-19, status as being quarantined during the COVID-19 pandemic, worry about COVID-19 infection, level of economic loss due to the COVID-19 pandemic, monthly income, regular physical exercise during the pandemic, presence of chronic physical illnesses, history of psychiatric disorders, and suicidality during the COVID-19 outbreak.

Insomnia symptoms were assessed with the validated Chinese version of the Insomnia Severity Index (ISI) questionnaire (34, 35), which consisted of seven items that assessed (1) severity of sleep onset problem; (2) sleep maintenance problem; (3) early morning wakening problem; (4) sleep dissatisfaction; (5) interference of sleep difficulties with daytime functioning; (6) noticeability of sleep problems by others, and (7) distress caused by sleep difficulties. Each item was rated on a 5-point Likert scale from “0” (no problem) to “4”(very severe problem). Total ISI scores ranged from 0 to 28, with a higher score indicating more severe insomnia symptoms. Following previous studies, a total score of ≥ 8 was considered as “having insomnia symptoms.” More specifically, a score between 8 and 14 was defined as “subthreshold insomnia,” a score between 15 and 21 was defined as clinical insomnia (moderate severity), and a score between 22 and 28 was defined as clinical insomnia (severe) (36, 37). The Chinese version of ISI had good reliability and validity in Chinese populations (38, 39).

Depressive symptoms were measured with the validated Chinese version of the nine-item Patient Health Questionnaire (PHQ-9) (40, 41). Each item was rated on a 4-point frequency scale from “0” (not at all) to “3”(nearly every day); total scores ranged from 0 to 27, with higher values indicating more severe depressive symptoms. Anxiety symptoms were measured with the validated Chinese version of the seven-item Generalized Anxiety Disorder scale (GAD-7) (42, 43). Each item was scored on a 4-point frequency scale from “0” (not at all) to “3”(nearly every day). Total scores ranged from 0 to 21 with higher overall scores indicating more severe anxiety symptoms. Global quality of life (QoL) was assessed by summing scores on the first two items on the Chinese version of the World Health Organization Quality of Life-Brief Version (WHOQOL-BREF) (44–46); a higher total score reflected better QoL.

### 2.3. Statistical analysis

#### 2.3.1. Univariate and multivariate analyses

Univariate and multivariate analyses were performed using SPSS version 26.0 (SPSS Inc., Chicago, Illinois, USA). Normality distributions of continuous variables were assessed using one-sample Kolmogorov-Smirnov tests and P-P plots. Comparisons of demographic and clinical variables including COVID-19-related variables between subgroups with and without insomnia symptoms were conducted using independent sample *t*-tests or Mann-Whitney *U* tests for continuous variables and Chi-square tests for categorical variables, as appropriate. Analysis of covariance (ANCOVA) was used to compare QoL between subgroups with and without insomnia symptoms after controlling for variables that had significant differences in univariate analyses. A standard binary logistic regression analysis was performed to examine independent correlates of insomnia status. Having insomnia symptoms was the dependent variable; measures on which there were significant group differences in univariate analyses were entered as independent variables. Significant statistical differences were set at *P* < 0.05 (two-tailed).

#### 2.3.2. Network structure

A network analysis was conducted using R software (47). In the insomnia network structure, nodes represented individual insomnia symptoms and edges represented the correlations between symptoms. Thicker edges represented stronger correlations, with green edges indicating positive correlations and red indicating negative correlations. To assess associations between different insomnia symptoms in the network model, a Graphical Gaussian Model (GGM) with graphic least absolute shrinkage and selection operator (LASSO) and an Extended Bayesian Information Criterion (EBIC) model were applied (48) to improve the accuracy of prediction, simplicity and interpretability of the network model (49). Network estimation was assessed using the “estimateNetwork” function in R “bootnet” with “EBICglasso” as the default method and the “qgraph” package for visualization (48). The “ggplot2” package was used for optimize visualization of the network (48, 50). Expected Influence (EI) was used to determine central symptoms in the network model in light of its reliability as a centrality index (51). Predictability was assessed using the “mgm” package (52) to calculate the variance in a node that could be explained by neighboring nodes in the network model (31). In addition, the “flow” function in R package “qgraph” was adopted to identify particular insomnia symptoms that were directly associated with QoL (53).

Stability and accuracy of the network model were evaluated using the R-package, “bootnet” (Version 1.4.3) (48). Stability was examined using the correlation stability coefficient (CS-coefficient), which calculates the maximum proportion of dropped cases to maintain a correlation above 0.7 between the centrality indices of the original sample and those of subset samples with a 95% probability (48). Following previous studies (17, 49), a CS-coefficient value above 0.25 was viewed as an acceptable stable network model, though 0.5 was preferable. Edge accuracy was estimated using bootstrapped 95% confidence intervals (CIs), with a narrower CI suggesting a more trustworthy network (48). A non-parametric bootstrapped difference test was performed to evaluate differences between edges pairs (48).

## 3. Results

### 3.1. Participant characteristics

A total of 1,020 Macau residents were invited to participate in this study, of whom 1,008 met entry criteria and completed the assessment for a participation rate of 98.82%. Demographic and clinical characteristics of the sample are shown in Table 1. The mean age of participants was 34.8 years (SD = 11.5 years) and 26.7% (*n* = 269) were male. Most participants had an education of college level or above (*n* = 831; 82.4%) and lived with other people (*n* = 935; 92.8%).

**TABLE 1:** Demographic characteristics of the study sample (*N* = 1,008).

Measure	Total (*N* = 1,008)		Without insomnia (*N* = 514)		With insomnia (*N* = 494)		Univariable analysis		
	*n*	%	*n*	%	*n*	%	χ^2^	*df*	*p*
Male	269	26.7	131	25.5	138	27.9	0.772	1	0.380
Married	471	46.7	254	49.4	217	43.9	3.049	1	0.081
Living with others	935	92.8	478	93.0	457	92.5	0.089	1	0.766
College and above education	831	82.4	433	84.2	398	80.6	2.349	1	0.125
Employed during the COVID-19 pandemic	687	68.2	349	67.9	338	68.4	0.032	1	0.859
Very concern about the COVID-19 pandemic	764	75.8	377	73.3	387	78.3	3.424	1	0.064
Being quarantined during the COVID-19 pandemic	109	10.8	41	8.0	68	13.8	8.752	1	**0.003**
Worried about COVID-19 infection							12.843	2	**0.002**
No worry	396	39.3	218	42.4	178	36.0			
Worried	462	45.8	239	46.5	223	45.1			
Very worried	150	14.9	57	11.1	93	18.8			
Economic loss							70.528	2	**<0.001**
Noor minimal	370	36.7	236	45.9	134	27.1			
Fair	405	40.2	211	41.1	194	39.3			
Very much	233	23.1	67	13.0	166	33.6			
Monthly income (≥MOP 30,000)	381	37.8	213	41.4	168	34.0	5.917	1	**0.015**
Physical exercise during the pandemic (≥30 min/day)	471	46.7	256	49.8	215	43.5	3.995	1	**0.046**
Presence of chronic physical diseases	34	3.4	14	2.7	20	4.0	1.357	1	0.244
Having a history of psychiatric disorders	58	5.8	11	2.1	47	9.5	25.259	1	**0.001**
Any suicidality during the latest COVID-19 outbreak	90	8.9	13	2.5	77	15.6	52.821	1	**<0.001**
	**Mean**	***SD***	**Mean**	***SD***	**Mean**	***SD***	***Z***	***df***	***p***
Age (years)	34.85	11.517	35.54	12.027	34.13	10.927	−1.428	—*	0.153
PHQ-9 total	7.32	6.058	4.15	3.843	10.61	6.187	−18.204	—*	**<0.001**
GAD-7 total	5.33	5.189	2.82	3.33	7.94	5.48	−16.534	—*	**<0.001**
Global quality of life	6.07	1.47	6.66	1.324	5.46	1.367	−13.462	—*	**<0.001**

Bolded values: <0.05. df, degree of freedom; PHQ-9, Patient Health Questionnaire-9 items; GAD-7, Generalized Anxiety Disorder-7 items; SD, standard deviation. 1 USD = 8.078 MOP; *Mann-Whitney *U* test.

### 3.2. Prevalence and correlates of insomnia

The overall prevalence of insomnia (ISI-7 total score ≥ 8) was 49.0% (*n* = 494; 95% CI = 45.9–52.1%). With respect to severity levels, 303 (30.1%) reported subthreshold insomnia, 147 (14.6%) reported moderate insomnia, and 44 (4.4%) reported severe insomnia. Table 1 summarizes differences between subgroups with and without insomnia. People with insomnia were more likely to be quarantined during the COVID-19 pandemic (*P* = 0.003), more worried about COVID-19 infection (*P* = 0.002), experienced economic losses during the COVID-19 pandemic (*P* < 0.001), have history of psychiatric disorders (*P* < 0.001), experience suicidality during the COVID-19 outbreak (*P* < 0.001), more likely to report having a lower monthly income (MOP < 30,000) (*P* = 0.015) and report less than 30 min of physical exercise every day during the pandemic (*P* = 0.046) when compared to people without insomnia. Additionally, those with insomnia reported higher mean levels of depression (PHQ-9) (*P* < 0.001) and anxiety (GAD-7) (*P* < 0.001) as well as a lower mean QoL score (*P* < 0.001).

After controlling for variables on which there were significant group differences in univariate analyses, the insomnia subgroup still had a lower QoL score (F_(1,1,008)_ = 17.450, *P* < 0.001) than did the no insomnia subgroup. The logistic regression analysis revealed that people with insomnia were comparatively more likely to report depression (OR = 1.237; *P* < 0.001), anxiety symptoms (OR = 1.119; *P* < 0.001), and being quarantined during the COVID-19 pandemic (OR = 1.172; *P* = 0.034) (see Table 2).

**TABLE 2:** Independent correlates of insomnia among Macau residents during the COVID-19 pandemic (*N* = 1,008).

Measure	Multiple logistic regression analysis		
	*p*	*OR*	95% *CI*
Being quarantined during the COVID-19 pandemic	**0**.**034**	1.172	1.043–2.811
**Worried about COVID-19 infection**			
No worry	-	–	–
Worried	0.161	1.269	0.910–1.769
Very worried	0.739	0.918	0.556–1.517
**Economic loss**			
No or minimal	-	–	–
Fair	0.300	1.199	0.851–1.690
Very much	0.065	1.537	0.973–2.428
Monthly income (≥MOP 30,000)	0.594	1.092	0.789–1.512
Physical exercise during the pandemic (≥30 min/day)	0.460	1.124	0.825–1.532
Having a history of psychiatric disorders	0.753	1.142	0.499–2.612
Any suidality during the latest COVID-19 outbreak	0.952	1.024	0.468–2.242
PHQ-9 total	**<0**.**001**	1.237	1.174–1.303
GAD-7 total	**<0**.**001**	1.119	1.059–1.184

Bolded values: <0.05. *CI*, confidence interval; *OR*, odds ratio.

### 3.3. Network structure of insomnia symptoms

**FIGURE 1:**
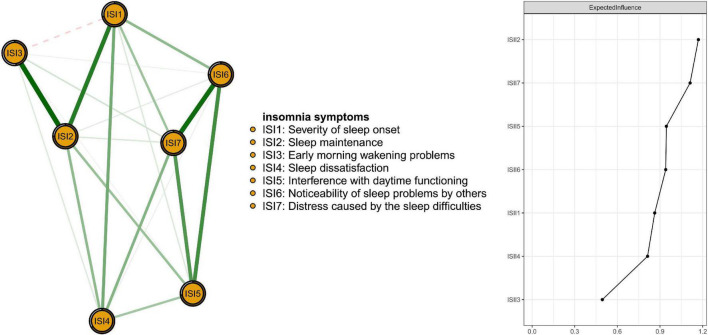
Network structure of insomnia symptoms among Macau residents during the COVID-19 pandemic.

Figure 1 presents the network structure of insomnia symptoms as measured by ISI items. The three nodes with the highest centrality were ISI2 (“Sleep maintenance problems”), ISI7 (“Distress caused by the sleep difficulties”), and ISI5 (“Interference with daytime functioning”). The mean predictability was 0.696, indicating an average of 69.6% of the variance in each node could be accounted for by its neighboring nodes in the model. Descriptive information and network centrality indices of each insomnia symptom are shown in Supplementary Table 1.

**FIGURE 2:**
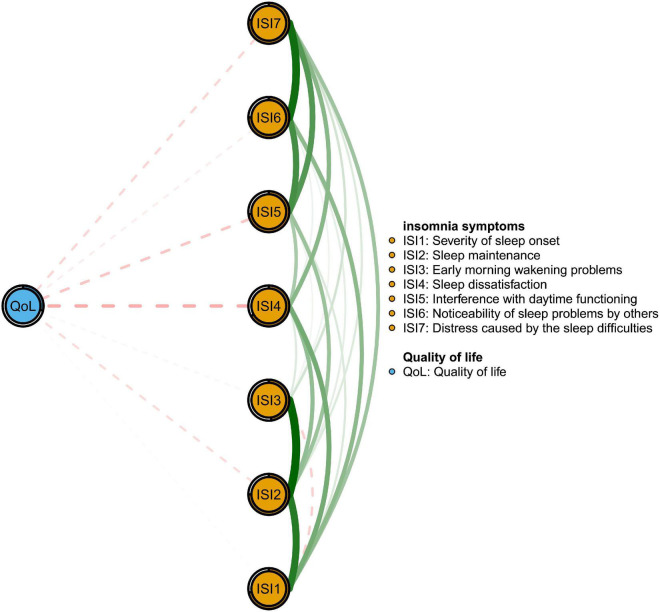
Flow network of quality of life and insomnia symptoms.

**FIGURE 3:**
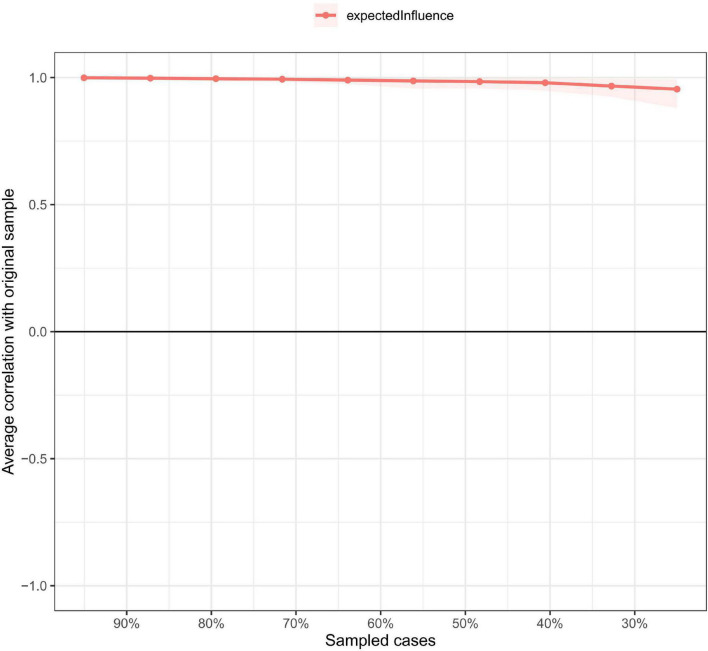
Network stability of insomnia symptoms among Macau residents during the COVID-19 pandemic.

Figure 2 shows that ISI4 (“Sleep dissatisfaction”; average edge weight = −0.13) had the strongest negative association with QoL, followed by ISI5 (“Interference with daytime functioning”; average edge weight = −0.09) and ISI7 (“Distress caused by the sleep difficulties”; average edge weight = −0.06). Figure 3 presents network stability results. The CS-C of EI was 0.75 based on the case-dropping bootstrap procedure, indicating that the network model was stable. For the accuracy of the network, bootstrap 95% CIs for estimated edge weights showed a narrow range (see Supplementary Figure 1); most of the edge weights were non-zero, suggesting that a majority of edges were stable and accurate. Bootstrapped difference tests for edge weights showed that most comparisons among edge weights were statistically significant, underscoring how the network model was reliable (Supplementary Figure 2).

## 4. Discussion

This was the first study to explore the epidemiology, correlates, and network structure of insomnia and its links with QoL among Macau residents shortly after the 618 COVID-19 wave.

Overall, the prevalence of insomnia was 49% (95% CI: 45.9–52.1%). Notably, this was much higher than the previous rate (27.6%; 95% CI: 24.8–30.4%) found among Macau residents during the first wave of the COVID-19 pandemic (17); as such, this finding suggests there may have been an exacerbation of sleep disturbances in this community. In addition, this rate exceeds rates reported in studies on the general population of mainland China (24.7%) (54), healthcare workers (34⋅3%; 95% CI: 27.5–41.5%) (55) and COVID-19 survivors (26.5%) (56). The high current rate in Macau may be due, in part, to the rapid and extensive transmission of the virus during this wave, the associated lockdown of most residents for the first time in over 2 years since the pandemic onset, and the suspension of daily activities. Together, these influences likely contributed to increased stress responses and disturbances in the sleep habits of many residents (57). One previous study has also found the severity of the pandemic and lockdown policies in Chinese cities are related to significant increases in rates of insomnia (58).

We also explored the network structure of insomnia at the symptom level in the sample. The node, “Sleep maintenance problems” (ISI2) was the most central symptom in the insomnia network model and indicated difficulties maintaining sleep (e.g., not sleeping well at night and waking up frequently) had the strongest connections with other symptoms in the insomnia network. Other influential nodes included “Distress caused by sleep difficulties” (ISI7), tapping residents’ worry/distress about current sleep problems, and “Interference with daytime functioning” (ISI5) due to insomnia. These findings align with a previous insomnia network model study in which “Interference with daytime functioning” (ISI5), “sleep maintenance” (ISI2), and “Distress caused by the sleep difficulties” (ISI7) were the most central symptoms of mental health professionals in China (29). Across both samples, these symptoms were most influential within insomnia network models and were more likely to predict insomnia than more peripheral symptoms (59). As such, concerns with sleep maintenance, distress caused by sleep problems and inference with daily functioning should be targeted for insomnia interventions while reductions of these symptoms may be key markers of treatment success. Furthermore, much of the variance of individual insomnia symptoms could be explained by this network model with an average predictability of 69.6%. This finding underscores strong inter-correlations between insomnia symptoms in this sample. The remaining variance may be attributed, in part, to other psychiatric disturbances related to insomnia, such as depression and anxiety (17).

Several correlates of insomnia among Macau residents also emerged in the study. Notably, people who were quarantined during this COVID-19 pandemic were more likely to suffer from insomnia, consistent with previous studies (57, 60). Although quarantine is an effective strategy for controlling transmission of the virus (61), it also limits resident zones of activity, usual routines and autonomy; such losses are related to negative mental health outcomes including depression, anxiety, insomnia, and suicidality (62). Previous studies have contended that stress induced by COVID-19 may lead to elevated levels of inflammation in socially isolated populations and result in mood and sleep disturbances (57). Long-time exposure to social media during isolation or quarantine could also disrupt the circadian rhythms of quarantined persons (63).

In addition, insomnia had significant positive correlations with depressive and anxiety symptoms in the sample. Previous evidence suggests that people with mood disorders and anxiety disorders have 3.3-fold and 2.6-fold risks, respectively, for developing insomnia compared to non-diagnosed controls (15). In another cross-sectional study, depression was more strongly related to sleep initiation difficulties while anxiety was more strongly linked to difficulty maintaining sleep (64). Close relations between these psychiatric disorders and insomnia may be due to overlapping neurobiology (65, 66) and psychosocial stressors that affect both sleep and emotional status (67). During this COVID-19 wave, the rising number of confirmed cases, continual dissemination in the daily news and mandated daily nucleic acid testing and/or rapid antigen self-testing might have exacerbated depression, anxiety symptoms, and insomnia (68).

In one recent depression-anxiety-insomnia network model study conducted during the pandemic, “sleep problems” emerged as a bridge symptom (17) linking these three psychiatric disorders. Indeed, possible bidirectional relations of anxiety and depression with insomnia are widely accepted and negative spirals in one or more of these disturbances could contribute to exacerbations in symptoms of other syndromes (15, 69, 70). From this perspective, interventions to alleviate insomnia problems such as cognitive behavioral therapy may help to address both sleep disturbances and comorbid disorders (71).

Concerning sociodemographic correlates of insomnia, neither age nor gender was significant. Previous studies reported women (15) and younger adults (72) were more likely to have insomnia symptoms. The discrepancy with past work may have reflected similar responses to the COVID-19 wave among different demographic groups or the larger proportion of women (73.3%) and young age (mean age = 34.8 years) of this sample. Insomnia was not significantly associated with regular physical exercise in the multivariate model for this sample in contrast to pre-pandemic evidence of a negative association (73). The null association in this study may have been a partial function of confinement and reduced opportunities for exercise among most residents during the “618” COVID-19 waves.

Economic losses and lower-income levels were associated with insomnia symptoms in univariate analyses but did not make unique contributions within the multivariate prediction model. Although previous studies have reported significant links between financial burdens and the severity of insomnia (74, 75), the relatively high level of economic security in Macau SAR may have had buffering effects. Despite significant economic losses from the COVID-19 pandemic, the gross domestic product (GDP) per capita in Macau versus mainland China (US$ 45,421.6 *versus* US$12,556.3) remained relatively high in the region and ranked 31^st^ in the world (76). The Macau SAR government also launched targeted financial support programs to promote economic recovery and guarantee free distribution of anti-epidemic supplies (e.g., KN95 masks and self-test COVID-19 antigen kits) for residents (8). These measures may have provided some relief from economic pressure. In addition, associations between economic factors and other correlates of insomnia status (depressive and anxiety symptoms) may have attenuated unique contributions of economic correlates in the multivariate prediction model.

The negative relationship between insomnia and QoL observed here has been found in other studies (77–79). In the flow network model, insomnia symptoms of “Sleep dissatisfaction” (ISI4), “Interference with daytime functioning” (ISI5) and “Distress caused by sleep difficulties” (ISI7) had the strongest, most direct negative associations with QoL and are plausible targets for reducing insomnia and improving QoL. Previous research suggests that sleep dissatisfaction is a significant predictor of sleep disorder severity among people with insomnia (80) and is regarded as a key indicator of sleep quality (81). Poor sleep quality (e.g., insufficient sleep) corresponds to poor physical or mental health outcomes including high blood pressure (82), cardiovascular diseases (83), depression (84) and suicidal behavior (85). In addition, poor sleep can deplete energy during the daytime and interfere with the daily functioning of samples assessed during the pandemic (77, 86). Maintaining a daily routine (e.g., work or studying) may play an important role in maintaining QoL (87). Furthermore, distress caused by insomnia such as nervousness or negative mood, and fatigue could affect perceived QoL (79). Once again, interventions to increase satisfaction with sleep also provide a viable pathway for improving QoL among insomnia sufferers.

The merits of this study included its large sample size and use of network analysis to pinpoint central symptoms of insomnia and those having stronger links to QOL. The main limitations should be acknowledged as well. First, this was a non-experimental, cross-sectional study, so causal relations between insomnia and other factors could not be drawn. Second, this study was conducted in Macau SAR, so results may be not representative of other regions due to different COVID-19 trajectories and policies. Third, to reduce the possibility of COVID-19 infection, an online survey based on snowball sampling was conducted; as a result, the representativeness and generalizability of results may have been reduced due to selection biases. Thus, the random sampling method should be used in future studies. Fourth, some factors related to insomnia, such as occupation and social support, were not recorded. More relevant variables should be included in future studies. Finally, because the assessment was based on self-reports, biases in recall and social desirability could not be controlled.

In conclusion, the comparatively high prevalence of insomnia among Macau residents during the recent COVID-19 wave and its association with poor QoL warrant attention. Being quarantined during the wave and concurrent elevations in symptoms of depression and anxiety were significant, unique correlates of insomnia. “Sleep maintenance” (ISI2) was the most central symptom in the network model of insomnia, while “Sleep dissatisfaction” (ISI4) had the strongest relationship with poor QoL. Future interventions should prioritize reducing problems in sleep maintenance as a means of improving insomnia and QoL.

## Data availability statement

The datasets presented in this article are not readily available because the Institutional Review Board (IRB) of the University of Macau that approved the study prohibits the authors from making publicly available the research dataset of clinical studies. Requests to access the datasets should be directed to Y-TX, xyutly@gmail.com.

## Ethics statement

The studies involving human participants were reviewed and approved by the Institutional Review Board (IRB) of the University of Macau. The patients/participants provided their written informed consent to participate in this study.

## Author contributions

LZ, SS, YF, and Y-TX: study design. PC, ML, K-IL, IC, TS, ZS, and TC: data collection, analysis, and interpretation. PC and Y-TX: drafting of the manuscript. TJ: critical revision of the manuscript. All authors approved the final version for publication.
