# Supplementary material for: Prevalence of insomnia and its association with quality of life among Macau residents shortly after the summer 2022 COVID-19 outbreak: A network analysis perspective
Source: Front Psychiatry. 2023 Feb 16;14:1113122. doi: 10.3389/fpsyt.2023.1113122 (PMC9978518; doi:10.3389/fpsyt.2023.1113122)

**Supplementary materials**

Table S1. Descriptive information and network centrality indices of insomnia symptoms

Figure S1. Bootstrapped confidence intervals of edge weights

Figure S2. Estimation of edge weight difference by bootstrapped difference test

Table S1. Descriptive information and network centrality indices of insomnia symptoms

| Item | Item content | Mean (SD) | Predictability | EI |
| --- | --- | --- | --- | --- |
| ISI1 | Severity of sleep onset | 1.17 (1.103) | 0.720 | 0.863 |
| ISI2 | Sleep maintenance | 1.20 (1.153) | 0.751 | 1.170 |
| ISI3 | Early morning wakening problems | 1.10 (1.090) | 0.481 | 0.494 |
| ISI4 | Sleep dissatisfaction | 1.84 (1.027) | 0.663 | 0.812 |
| ISI5 | Interference with daytime functioning | 1.20 (1.102) | 0.728 | 0.944 |
| ISI6 | Noticeability of sleep problems by others | 0.97 (1.069) | 0.751 | 0.910 |
| ISI7 | Distress caused by the sleep difficulties | 1.04 (1.099) | 0.776 | 1.112 |

Note: SD: standard deviation; EI: Expected influence.

Figure S1. Bootstrapped confidence intervals of edge weights


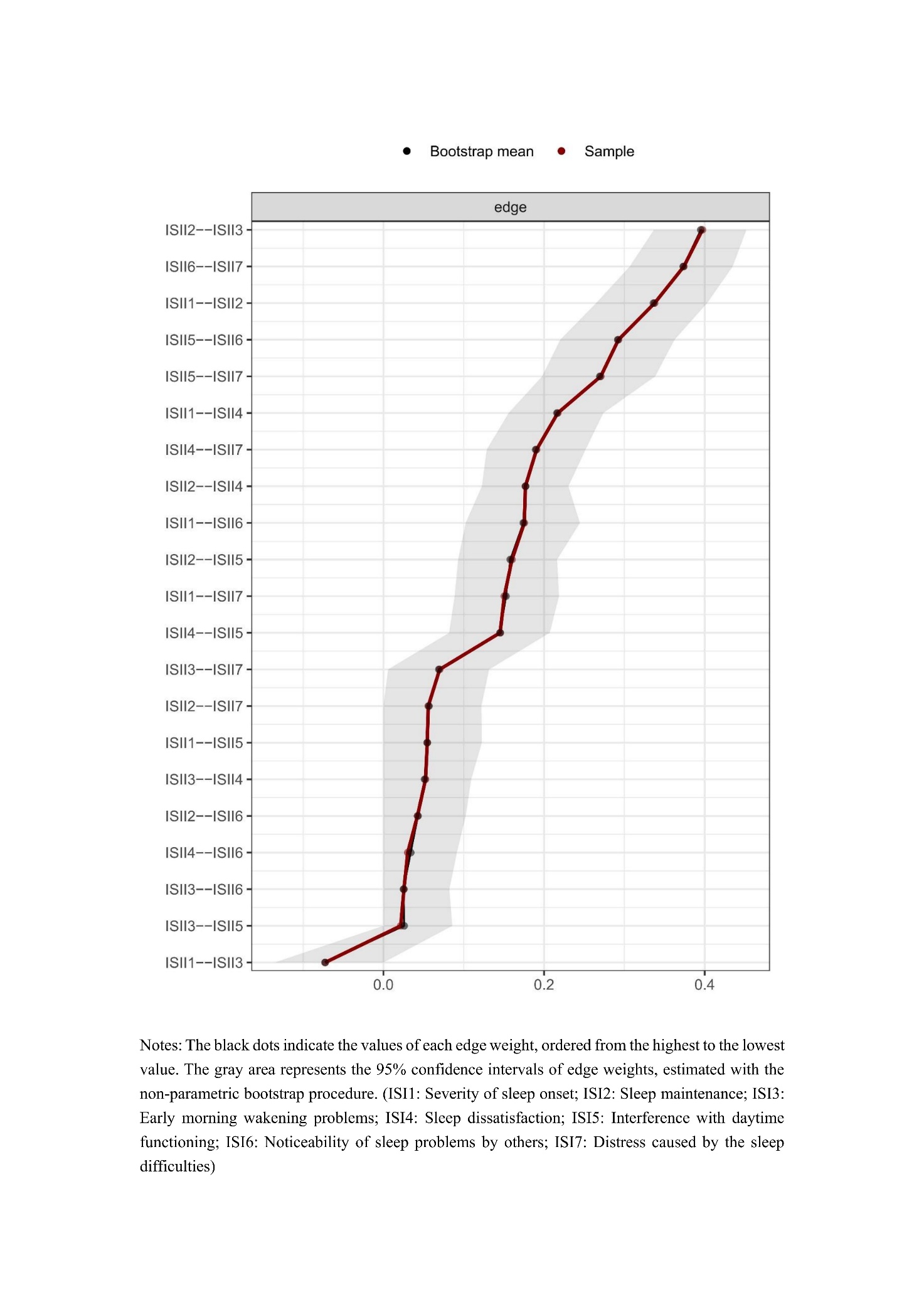


Figure S2. Estimation of edge weight difference by bootstrapped difference test


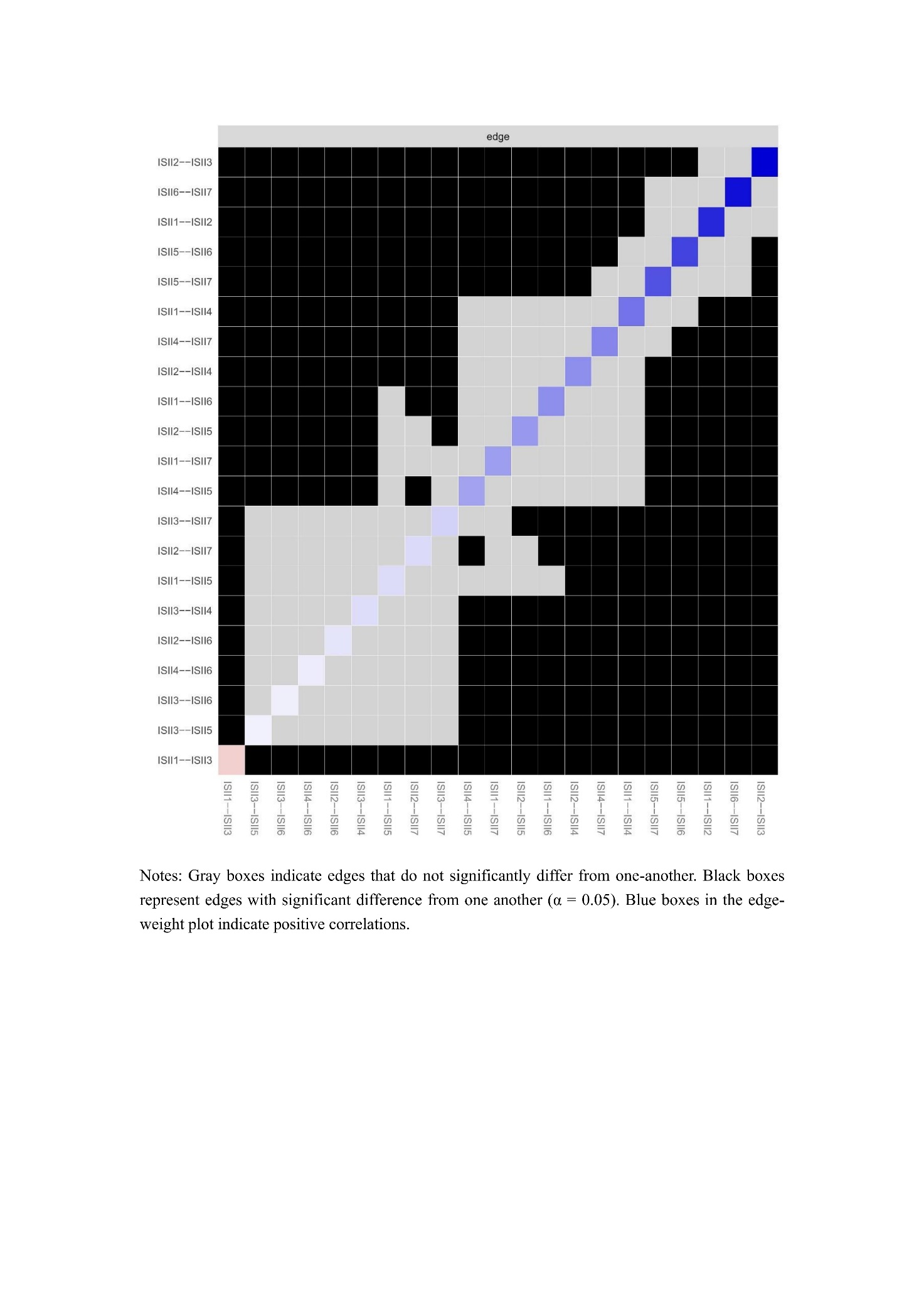

Supplement: Supplementary file 1 [file Data_Sheet_1.docx]
